# Influence of macular pigment on the sensitivity to discomfort glare from daylight

**DOI:** 10.1038/s41598-023-45785-x

**Published:** 2023-10-29

**Authors:** Sneha Jain, Jan Wienold, Chiara Eandi, Sara Gisselbaek, Aki Kawasaki, Marilyne Andersen

**Affiliations:** 1 Laboratory of Integrated Performance in Design (LIPID), École Polytechnique Fédérale de Lausanne (EPFL), 1015 Lausanne, Switzerland; 2 Hôpital Ophtalmique Jules-Gonin, Lausanne, Switzerland; 3 Faculty of Biology and Medicine, Université de Lausanne (UNIL), Lausanne, Switzerland

**Keywords:** Civil engineering, Human behaviour, Psychology and behaviour, Eye manifestations, Quality of life

## Abstract

Understanding the factors that influence the human perception of glare is necessary to properly address glare risks in buildings and achieve comfortable visual environments, especially in the workplace. Yet large inter-individual variabilities in glare perception remain unexplained and thus uncovered by the current empirical glare models. We hypothesize that this variability has an origin in the human retina, in particular in the density of macular pigments present in its central area, which varies between individuals. Macular pigments are known to absorb blue light and attenuate chromatic aberration, thus reducing light scatter. This study presents the outcomes of the first experiment ever conducted in a daylit office environment, in which glare sensitivity and macular pigment density were measured and compared for 110 young healthy individuals, along with other ocular parameters. The participants were exposed to different glare conditions induced by the sun filtered through either color-neutral or blue-colored glazing. In neutral daylight conditions with sun disc in the near periphery, neither macular pigment nor any other investigated ocular factors have an impact on discomfort glare perception whereas glare perception in conditions with the blue-colored sun disc in the near periphery was found to be correlated with macular pigment optical density.

## Introduction

Designing buildings that facilitate optimum utilization of daylight is desirable for its many benefits including occupant well-being, productivity, energy conservation, and building sustainability. Nevertheless, it remains a source of light challenging to manage, given its dynamics and its associated over-heating and glare risks^1,2^. Excessive brightness from daylight in workplaces can indeed lead to discomfort glare which, if prolonged or recurrent, might reduce occupant well-being, mood, and performance^3,4^.

Discomfort glare causes visual irritation or annoyance without necessarily impairing the vision^5^. It has been studied for several decades and the prediction models resulting from the conducted user assessment studies are nowadays able to provide a reasonable estimate of visual discomfort for a group of observers^6^. However, glare models often perform poorly when it comes to individual comfort due to the large inter-individual variability in glare perception^7^. Since the physiological origin associated with discomfort glare mechanisms is not well understood, the factors responsible for the variability in the users’ perception cannot be included in current glare models. Existing glare models only account for various physical/geometrical factors related to the light source(s) present in one’s field of view. However, before potentially generating discomfort, light energy must first convert to the neural signals and this process occurs in the eye itself. Therefore, to improve glare prediction models, it would be worth investigating certain anatomic-physiologic features of the eye which are likely to influence glare perception based on available literature and thus generate variance between individuals.

One such ocular factor, most studied in the ophthalmology literature, is the density of yellowish macular pigments present in the fovea (central depression in retina with greatest visual acuity). Individuals with higher macular pigment density were reported to have better visual performance and higher tolerance to glare from electric light sources close to the fovea^8–13^. Macular pigment (MP), which is greatest in the foveal region (spread across 6.7° horizontal, 3.0° vertical) and decrease exponentially with increasing eccentricity from the centre of the fovea^14,15^, have absorption spectra between 400 to 550 nm, peaking at around 460 nm i.e. in the blue range of the light spectrum^16^. MP acts as a filter for short-wavelength light and reduces the scattered light reaching the photoreceptors. Its impact on blue light attenuation is measured by the Macular Pigment Optical Density (MPOD), which is linearly related to the amount of dietary carotenoids present in the macula^14^. All the previous studies on glare and MP relationships measured MPOD using the heterochromatic flicker photometry (HFP)^17^ method on a scale of 0 to 1, where higher values indicate denser pigments and better attenuation of blue light hitting the macula. There exists a wide variability in the amount of macular pigments across the population, therefore, causing a large variation in the amount of short-wavelength light processed by the retina^18^. Based on this, we hypothesized that the existing variability in sensitivity to glare could be partly caused by the variability in macular pigment density.

Several studies originating from the field of medical sciences have indicated that MP reduces glare by absorbing shortwave light in central vision. A study by Stringham et al.^8^ indicated that participants with higher MP levels had significantly better visual performance in glare conditions. Another study from the same authors^11^ showed that the participants with the broader spatial distribution of MP had higher visual discomfort thresholds which were further confirmed by Wenzel et al.^12^. Hammond et al.^9^ showed a significant contribution of MP in protection against disability and discomfort glare. A recent study by Wilson et al. showed that individuals with significantly higher MPOD levels experienced less eye pain or fatigue in their day-to-day activities assessed via questionnaires^10^. A few studies have also assessed the impact of MP on disability glare and photostress recovery time (which is the time taken to reach normal acuity after bleaching of photoreceptors by bright light) and found similar results^8,9^. However, Loughman et al.^19^ concluded that the visual performance under glare conditions was unrelated to macular pigments. The authors discussed that the absence of a strong blue light component (absorbed by MP) in their white LED light source, might be the reason for their findings being different than the other studies. Although, a later study by Stringham et al. also used white LEDs with lesser blue content but still found that MP influenced glare sensation^8^. Given the significant findings demonstrated by many studies, macular pigment remain a promising factor that can potentially influence glare perception.

Most of the discussed studies were conducted under ophthalmological laboratory settings, with a light source projected on the retina within 1° to 6° from the eye, to measure the glare sensitivity in foveal and parafoveal regions where MP is concentrated^11,12,20,21^. Additionally, most of the studies used a Maxwellian-view optical system with a xenon lamp that has a spectrum close to solar spectra with higher emission in the shortwave region. These conditions differ significantly from realistic settings and thus limit the applicability of the findings to the normal indoor environment. It is indeed not known whether the influence of MP would persist in normal working conditions where glare sources do not usually lie in the fovea, occupants have unrestricted gaze behaviour, and where the main source of glare has a broader spectrum, such as in the case of daylight from windows. Such investigations can provide complementary answers to the studies discussed above, with more direct applicability to the larger context of indoor workplace environments.

Towards this end, this study aims to determine the influence of MPOD on the discomfort glare perception under daylight conditions in an office-like setting where the sun visible through the window act as a main glare source.

We present the findings from two user experiments each with 55 healthy participants (age: 18–35 yrs.): one with color-neutral glazing (experiment-I) and another with blue-colored glazing (experiment-II). We measured participants’ MPOD levels and compared them with their glare perception assessed psychophysically by exposing them to four varying levels of sun intensity visible behind the glazing in each experiment. In experiment-I, test conditions had color-neutral glazing that differed in glazing transmittance whereas in experiment-II, conditions differed in glazing color (blue, green, red, neutral, a set that was designed as part of another study). The present analysis will be restricted to glare evaluations based on the full set of color-neutral scenarios from experiment-I and only on the blue glazing scenarios from experiment-II. Blue glazing was chosen since the light absorption by MP is the highest in the short-wavelength region whereas color-neutral glazing was chosen to represent regular office environments. In experiment-I, all participants took part in an additional two-hour long session at the ophthalmic hospital where relevant functional and structural eye exams were conducted for assessing their macula and ensuring a normal fundus without any retinal pathologies.

The primary objective of the study is to determine the influence of macular pigment on glare sensitivity under daylight as the only source of lighting and the secondary objectives are to determine the influence of functional and structural parameters that links to macular health, mainly the photostress recovery time and retinal thickness on glare sensitivity. To the best of our knowledge, this is the first-ever study that investigates the influence of MPOD on glare sensitivity under natural lighting conditions.

## Results

### Ocular measurements of the participants

Table 1 provides a descriptive summary of all the measured ocular parameters. The MPOD levels of the participants’ left and right eyes were measured in both experiments using screener device based on HFP. In experiment-I, we also measured the participants’ visual acuity at low contrast, their photostress recovery time, and their mean iris thickness. In addition, fundus photographs of both eyes were obtained and assessed qualitatively by an ophthalmologist to exclude the participants with fundoscopic-evident retinal pathologies.

**Table 1:** Summary of measured ocular parameters of the participants.

Experiment	Tests	Mean	Median	Minimum	Maximum	Std. dev.
Experiment I	MPOD	0.49	0.48	0.14	0.86	± 0.17
	PSRT (in seconds)	11.7	11	5	34	± 6
	Iris thickness (in μm)	278	280	247	321	± 19
	Acuity at low contrast	28	28	5	39	± 6.6
Experiment II	MPOD	0.47	0.45	0.19	0.82	± 0.16

**Figure 1:**
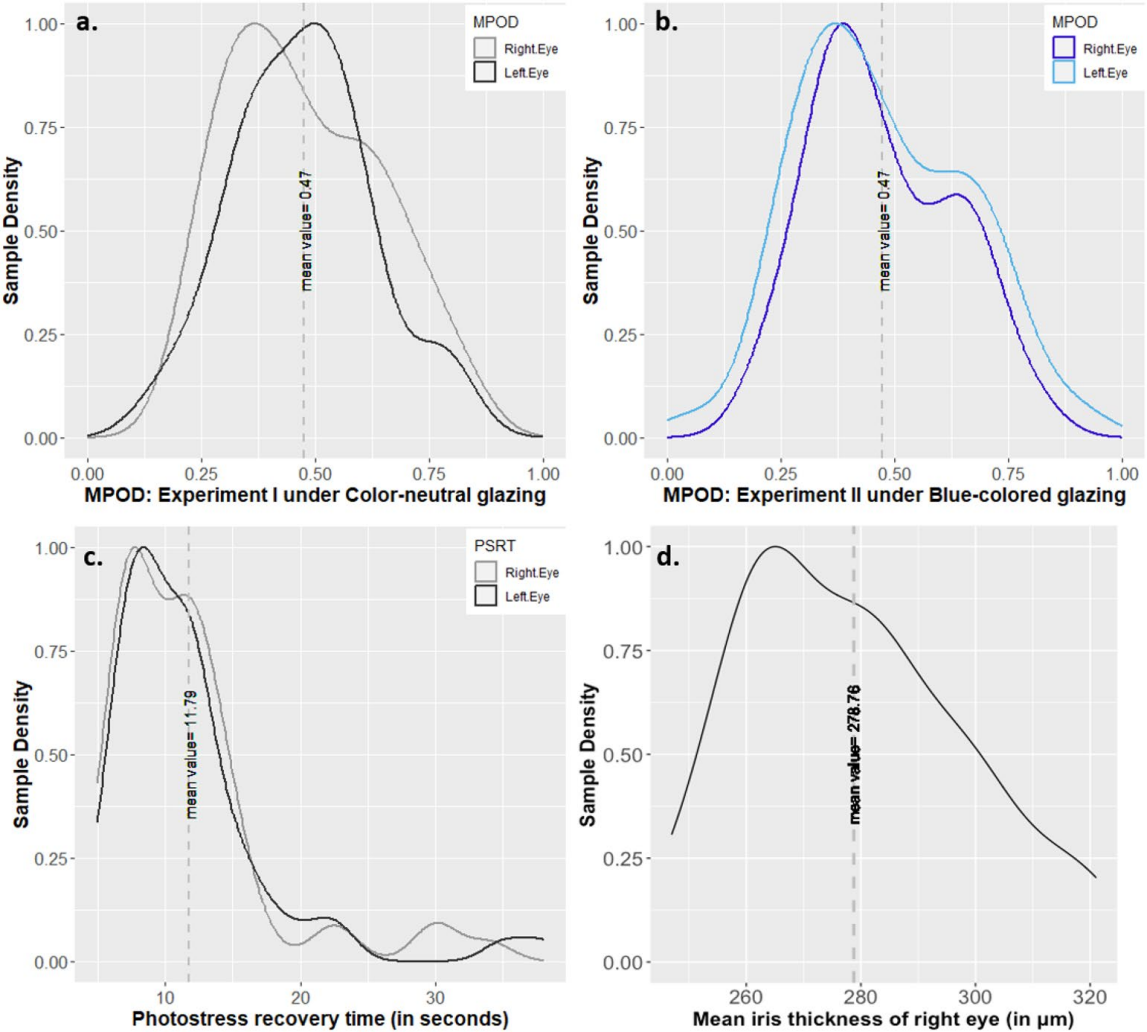
Density plots of measured ocular parameters of the participants: (**a**) MPOD values of participants’ eyes in Experiment I and (**b**) Experiment II, (**c**) Photostress recovery time and d: mean iris thickness of the participants’ right eye in experiment I.

Figure 1a and b show the distributions of the MPOD at 0° eccentricity for a centrally fixated target. It should be noted that the participants were not repeated between the experiments I and II, however, their age distribution were kept similar for comparison purpose (Table 5). We applied normality tests which confirmed that the data follows a normal distribution (Shapiro–Wilk test, p > 0.05) despite a slight positive skewness (0.29). From Table 1 and Fig. 1a and b, we can infer that the MPOD distributions between experiments I and II are similar (Kolmogorov–Smirnov test: D = 0.06, p = 0.99), with a mean value of 0.49 and 0.47, respectively. The MPOD ranges observed in our study are similar to previous studies with young and healthy adults^10,19,22^. The correlation coefficient between the MPOD measurements of the right and left eye is 0.87 (Pearson correlation-coefficient, p < 0.0001), indicating excellent inter-eye predictability of the measurement device. MPOD value minimum of OS (left eye) and OD (right eye) for each participant was selected for the analysis.

Figure 1c and d provide density plots for the participants’ photostress recovery time and mean iris thickness measured in experiment-I, respectively. A quicker photostress recovery time have been linked to denser macular pigment and higher visual discomfort thresholds^8^. From the plot, we can observe a mean recovery time of 11.7 s (ranging from 6 to 38 s). These values are consistent with prior studies in which recovery times of up to 40 s were observed among healthy individuals^23,24^. Iris thickness distribution, on the other hand, was measured using OCT images of the retina captured at three angles (temporal, nasal, and inferior), in each of which iris thickness was measured at 3 points to extract an overall mean value. The OCT images through the anatomical center of the macula were qualitatively examined to confirm of normal foveal depression profile among the participants and cases where a relative absence of foveal depression was found, were removed since it can potentially affect the health of the macula. Finally, low-contrast visual acuity was measured using precision vision charts with letters at 2.5% contrast as a baseline measure to ensure normal ocular health as reported in Table 1. These measured ocular data are further analyzed with subjective glare sensitivity in upcoming sections.

### Discomfort glare evaluations

#### Photometric and spectral characteristics of the experimental conditions

**Figure 2:**
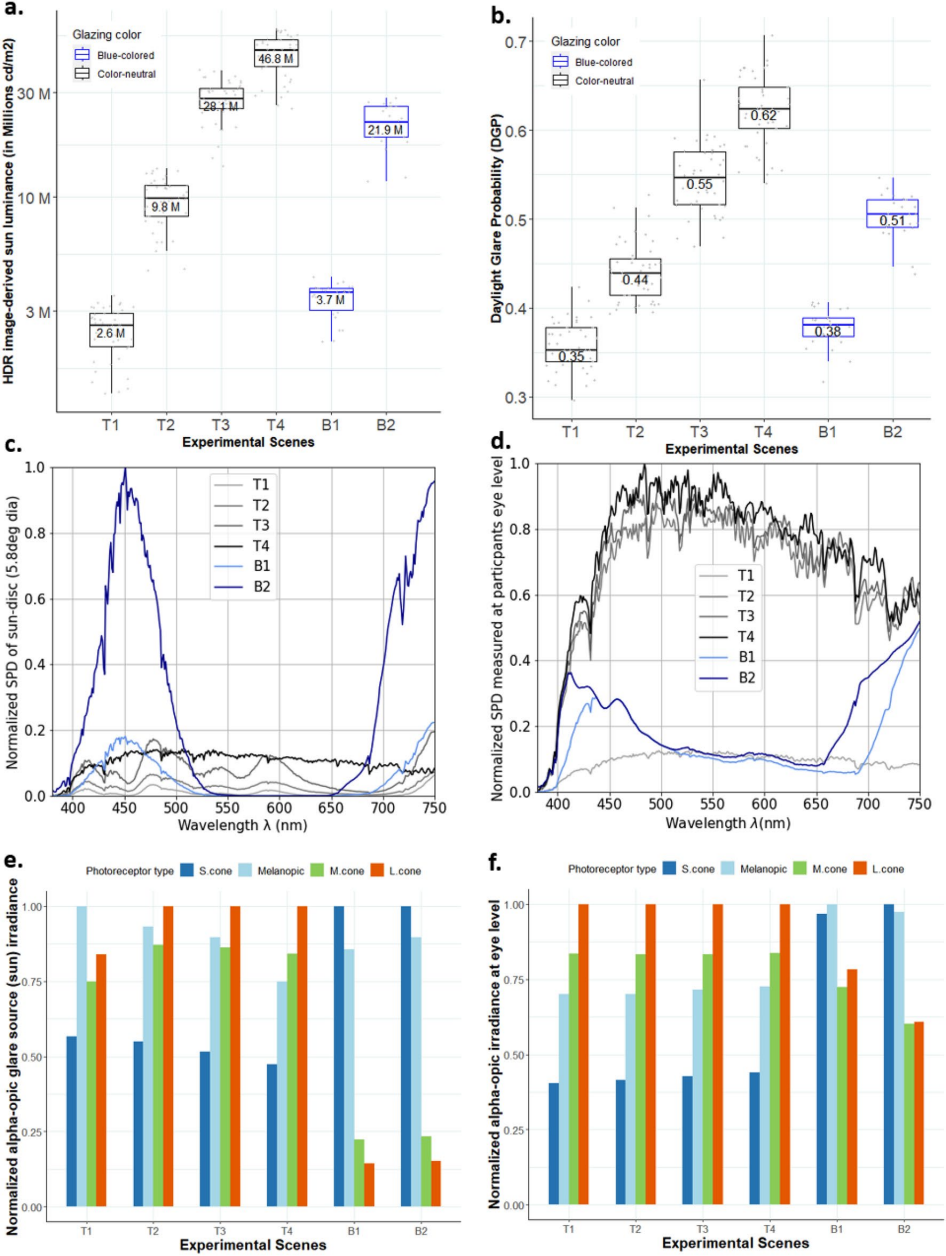
Measured photometric and spectral characteristics of daylight during the experiments. (**a**) Sun luminance (cd/m^2^) and (**b**) DGP values measured from HDR images (taken at participants’ eye level) shown as the boxplots with median values for each experimental condition. (**c**) Mean normalized SPDs of the sun disc (dia 5.8°) and (**d**) the SPDs of daylight measured at participants’ eye level for each condition. (**e**) Mean alpha-opic irradiance values calculated for the sun-disc (dia ~ 5.8°) and (**f**) calculated at the eye level for cones and ipRGCs photoreceptor types normalized over each experimental condition.

Each participant was exposed to four glare conditions (T1, T2, T3, and T4 in Table 2 and Fig. 6) for approximately 15 min in randomized order under color-neutral glazing in experiment-I. These four conditions differed in the transmittance of the glazing from which the sun was visible to the participants (“sun window” in Fig. 5). Similarly, in experiment-II, we created two glare conditions that differed in the transmittance value of the blue-colored glazing (B1 and B2 in Table 2 and Fig. 6) but each participant was exposed to only one of the two conditions for 15 min since the transmittance was varied between the participants. In both the experiments, daylight was the only source of lighting without any supplementation from electric light. In this section, we discuss the photometric properties of each condition shown to the participants to confirm that within each experimental condition, there was a low enough variance to consider that all participants were exposed to similar discomfort glare levels within a given glazing scenario. Discomfort glare is usually quantified based on contrast and amount of light in observers’ field of view^5^. We compared glare conditions shown to participants with all three types of glare metrics (Table 2, Fig. 2a and b): contrast driven metric- CGI (CIE Glare Index)^25^, amount of light or adaptation based metrics- E_v_ (Vertical illuminance at eye), hybrid metric based on both contrast and amount of light- DGP (Daylight Glare Probability)^26^.

**Table 2:** Summary of the descriptive statistics pertaining to all experimental conditions.

Experiment	Experiment I (color-neutral glazing)				Experiment II (blue-colored glazing)	
Scene	*T1*	*T2*	*T3*	*T4*	*B1*	*B2*
Sample size (after filtering)	45	45	44	45	25	25
Glazing τ_v_	0.36%	1.25%	3.40%	4.80%	0.39%	2.25%
Mean E_v_ (lux)	1,770	2,200	3,300	4,800	1,130	2,300
Mean sun luminance (millions cd/m^2^)	2.6	9.8	28	46	3.6	21.2
Mean DGP	0.35	0.44	0.55	0.62	0.38	0.5
Mean CGI	36.3	43.4	49.7	51.1	38.2	45.6
Mean position index	3.3	3.3	3.3	3.3	3.2	3.3
Mean viewing angle to the sun	32°	32°	32°	32°	31°	32°
Mean veiling luminance due to sun (in cd/m^2^)	1.1	4.8	14.8	24.5	2.6	16.5
Mean retinal illuminance (in Log Troland)	4.22	4.33	4.52	4.68	4.05	4.35

Table 2 presents a summary of the data collected during the two experiments conducted on sunny days with stable weather. These include data derived from High Dynamic Range (HDR) images that were captured at the participant’s eye level and processed to derive glare source (sun) luminance, E_v_, DGP, CGI, position index of the sun, the viewing angle between the sun and the observer, veiling luminance and retinal illuminance. They also include measured visible light transmittance (τ_**v**_) of the glazing from where the sun was visible to the participants (“sun window”). It can be observed from Table 2 that the position index and the viewing angle between the sun and the observer have similar mean values across experimental conditions in two studies, which indicates that we were successful in maintaining similar sun position in participants’ field of view. The mean values of E_v_, sun luminance, DGP, and CGI of the test conditions are increasing from T1 to T4 and B1 to B2 due to the increase in the glazing transmittance (τ_**v**_) allowing more daylight into the space. Mean veiling luminance from the sun as shown in Table 2 (calculated as per CIE report on disability glare^27^) indicate the intraocular light scatter, which is minimal in all the test conditions (1.1 to 24.5 cd/m^2^) and therefore, have minimal impact on reducing retinal image contrast and creating disability glare^28^. To further ensure that participants’ vision was not impaired due to the exposure to relatively higher sun luminance values, we implemented a computer screen based Landlot-C contrast test^29^. Participants did the contrast test under constant electric light setting without any daylight glare in the introduction phase as a baseline measure; afterwards, they repeated the same test (with randomized characters) under each daylight experimental condition. We compared the results of the baseline test with the test conducted under daylight and did not find any significant differences in their contrast sensitivity measures.

Figure 2a shows the distribution of sun-disc luminance (cd/m^2^) as boxplots for four conditions under color-neutral glazing and two conditions under blue-colored glazing. As expected, the sun's luminance increases as a function of window transmittance and that the luminance conditions experienced for each scene overlap very little, which was our goal. This indeed ensured that participants were exposed to different levels of glare, thereby consolidating the relative assessment of their discomfort glare thresholds.

Figure 2b presents the distribution of DGP values, which again increases as a function of window transmittance since the increased intensity of the sun disc elevates both the amount of light and contrast in the field of view. The DGP cut-off value, used to distinguish between disturbing and non-disturbing glare used in the European daylight standards for buildings EN17037, is 0.40^30^. This value categorizes all our experimental conditions as creating disturbing glare for the majority of the people, except the conditions T1 and B2 where the sun window transmittances were 0.36% and 0.39%, respectively.

Figure 2c and d present the mean normalized spectral power distribution (SPD) of the sun-disc (dia ~ 5.8°) and the SPDs measured at eye level for each experimental condition, respectively. As expected, the SPD of the sun follows the ‘sun window’ spectral transmittance curve shown in Fig. 7. While the SPDs at eye level (Fig. 2d) differ substantially from the sun-discs’ SPDs (Fig. 2c), particularly in case of blue-glazing. This is due to the contribution of daylight coming through the remaining color-neutral windows (Fig. 5), resulting in an attenuation of relative quantity of blue light reaching the eye.

Figure 2e and f demonstrate the mean alpha-opic (cones and ipRGCs photoreceptors) irradiance of sun-disc and of daylight reaching participants’ eye level, respectively, normalized for each experimental condition. These values are calculated from the SPDs shown in Fig. 2c and d, following the CIE guidelines^31^. In both the figures, blue-glazing conditions (B1 and B2) have relatively higher contributions of S-cone-opic and melanopic equivalent irradiance compared to M-cone-opic and L-cone-opic irradiance, unlike color-neutral conditions (T1 to T4). We further discuss the potential impacts of such contributions on participants’ glare sensitivity in the following section.

#### Participant’s subjective responses

**Figure 3:**
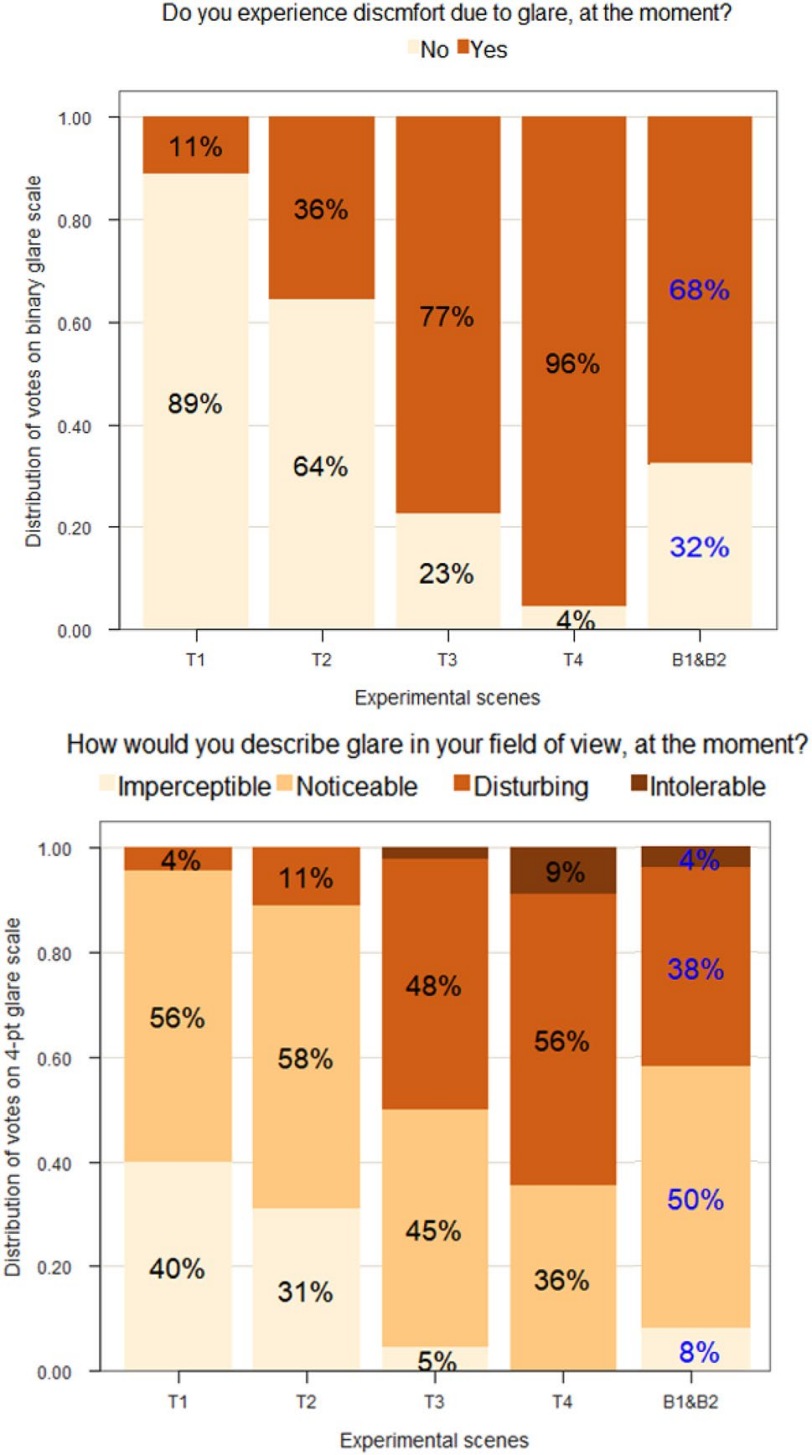
Distribution of participants’ responses to discomfort glare: on binary response labels (top) and on Likert four-point labels (bottom).

Participants performed a typing task while exposed to each test condition described in previous section and then answered specific questions (see Table 6) about their visual comfort. This section presents the distribution of their responses to glare questions in the form of stacked bar plots. Figure 3 shows the percentage distribution of glare responses from the participants, rated on a binary ‘’Yes/No” scale (question 2, Table 6) and on a four-point scale “Imperceptible/Noticeable/ Disturbing/Intolerable” (question 3, Table 6), respectively. Comparing the two glare questions in Fig. 3, a similar trend of glare voting can be seen, indicating an agreement between the participants’ responses. Furthermore, Cronbach’s alpha between questions 3, 4, and 5 in Table 6 is 0.93, showing an excellent inter-consistency between the participants’ answers^32^. In parallel, the answers to question 6 in Table 6, which pertained to eye fatigue, also observed a similar trend, i.e., participants’ eye fatigue increased with their discomfort from glare. Additionally, we also compared the results from the glare sensitivity test conducted in electric lighting (procedure mentioned in “Method” section) to the glare perception reported in daylight for each participant and found them consistent with each other (r = 0.71). Therefore, we decided to focus the analysis only on the glare responses received to questions 2 and 3 from Table 6, which are in fact also often used questions in many past discomfort glare studies^6,33,34^.

In Fig. 3, we can observe that, as expected, a greater number of participants experienced discomfort from glare when the window transmittance increased. The spearman’s rank correlation coefficient between the glare responses on the four-point scale and the DGP model is 0.62, indicating a strong correlation following Cohen’s effect-size thresholds^35^. However, the DGP and CGI thresholds under color-neutral daylit conditions are consistently higher than blue-colored conditions. In other words, participants experienced glare more strongly under the blue-colored sun, which follows previous literature^36^. Additionally, Fig. 2f shows that in blue-colored conditions, melanopic stimulation was higher than cone photoreceptors compared to color-neutral conditions which could be potentially related to the higher glare perception under blue-colored conditions. It also follows the findings from a recent study suggesting the role of melanopsin signals in the estimation of perceived brightness and consequently perceived glare^37^.

In color-neutral conditions of experiment-I, it can be seen in Fig. 3 that a majority of the participants switched their votes to “yes”, and to “disturbing” glare, when going from condition T2 (mean DGP 0.44) to T3 (mean DGP 0.55): the glare thresholds of reporting ‘disturbing’ glare under color-neutral scenarios are thus higher than the ones reported in the European daylight standards for buildings EN17037 (DGP threshold 0.40), indicating more tolerance to glare than what the DGP model would have predicted. We can also observe a rather high inter-individual variability among the participants’ responses when experiencing similar lighting conditions, which once again points to different levels of sensitivity towards discomfort glare from one person to another. From the available data, we decided to group participants who answered ‘Yes’ to question 2 (Fig. 3, top) as more sensitive towards glare, and the rest of the participants as being less sensitive to glare to compare their MPOD levels.

### Influence of MPOD on glare perception

**Figure 4:**
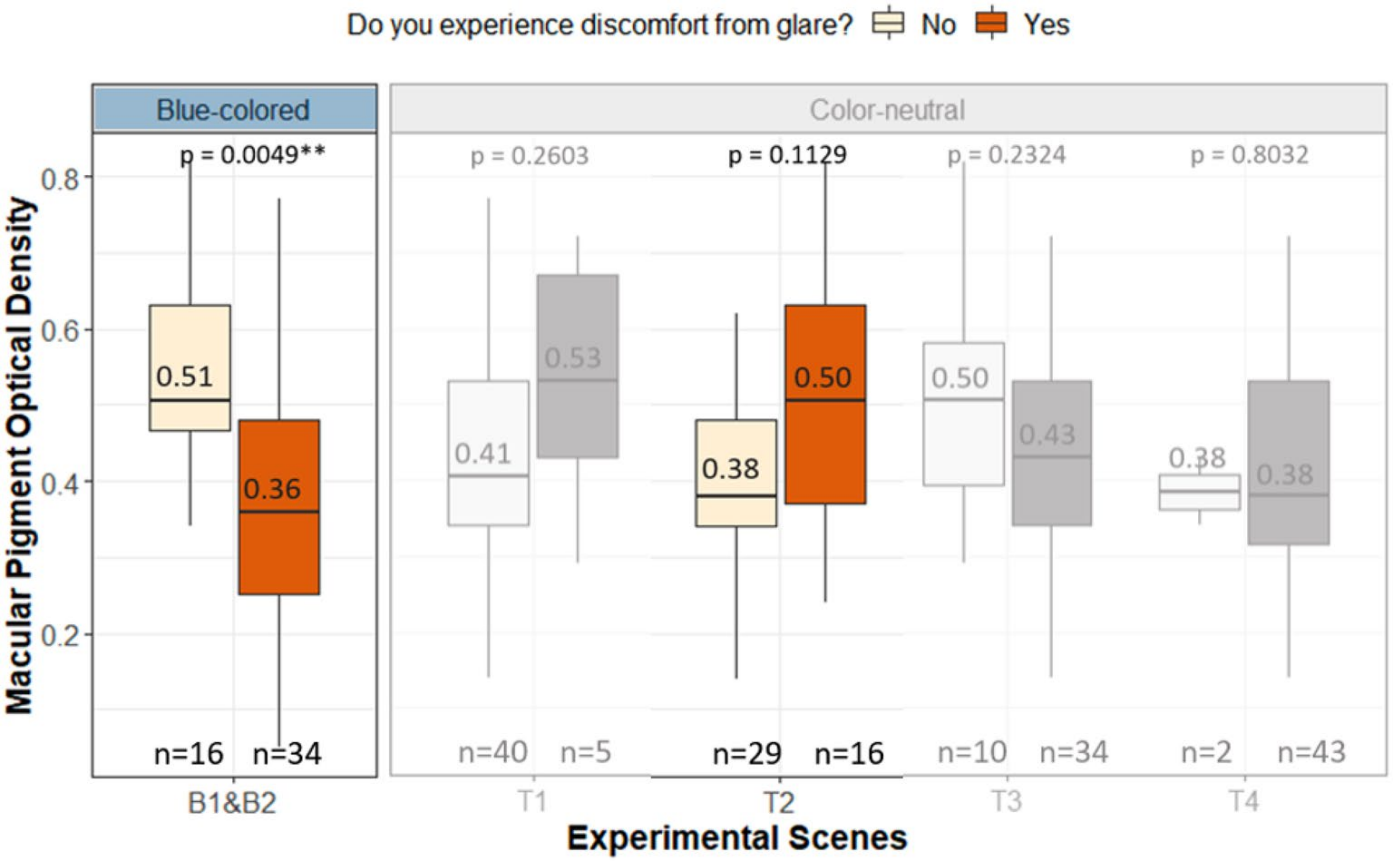
Box plots comparing the mean differences of MPOD between the two groups of participants less sensitive and more sensitive to glare under blue-colored and color-neutral glazing.

Based on the participants’ responses in both the experiments across all conditions and their inferred sensitivity to glare as explained above, we grouped them as more versus less sensitive to glare and then compared the measured MPOD levels between the two groups as shown in Fig. 4. We applied Wilcoxon signed-rank test^38^ to check whether the differences in MPOD levels between the more sensitive and less sensitive groups are statistically significant (Fig. 4 and Table 3).

**Table 3:** Results of the Wilcoxon rank-sum test assessing the statistical significance of the MPOD differences between the glare-sensitive and non-sensitive groups.

Response group	Experiment	Experimental scenes	Wilcoxon, p-value (Bonferroni corrected)	Effect size
Yes/No	Experiment I (color-neutral glazing)	T1	0.260	0.17
		T2	0.113	0.24
		T3	0.2324	0.18
		T4	0.8032	0.04
	Experiment II (blue-colored glazing)	B1 and B2	0.0049**	**0.40**

Statistically significant values are in bold.

None of the groups under color-neutral daylit conditions have statistically significant differences (at α = 0.05) in MPOD levels under all the experiment conditions (Fig. 4). In other words, variance in MPOD values is not an indicator of whether or not participants were sensitive to glare under neutral daylight. Since the number of data points in one of the two groups under T1, T3 and T4 conditions are either unbalanced or very low (n ≤ 5) which can make the results less reliable due to the test statistics not properly following a χ^2^ distribution^39^. We thus focused on the condition T2, as highlighted in Fig. 4, which has sufficient datapoints to draw results, however, we did not observe a statistically significant effect (p = 0.113) of MPOD on the participants’ glare sensitivity under color-neutral daylit scenarios.

In contrast to color-neutral conditions, we observed a significant difference (p-value = 0.0049, effect-size = 0.40: moderate) in the MPOD levels associated with each group exposed to the blue-colored sun-disc. Participants more sensitive to glare were found to have lower MPOD levels than the participants less sensitive to glare under blue-glazing conditions (Fig. 4). Participants with denser MP were better able to tolerate the glare from the blue-colored sun-disc. The median MPOD was 0.51 for less sensitive group where 0.36 for more sensitive group. Though, compared to past studies, here the glare source is not close to the fovea where MP is most concentrated. Therefore, we can hypothesize that in our study participants’ free gaze behaviour, unlike past studies, might have caused instances where the sun was in fact close to the fovea.

Similar findings came from the correlational analyses between MPOD and glare perception, presented in Table 4. The correlation coefficients are again shown as being significant with a moderate effect size only in the case of blue-colored daylit scenarios, while they remain non-significant in color-neutral scenarios.

**Table 4:** Correlation coefficients between participants’ subjective responses to glare and their measured ocular parameters.

Subjective response type (cf. Table 6)	Correlation metric	MPOD		Photostress recovery time	Iris thickness
		Blue	Neutral		
Binary (question 2)	Point-biserial	− 0.40*	0.27	0.04	0.22
4-point (question 3)	Spearman’s rank	− 0.38*	0.28	0.08	0.21
4-point eye strain (question 6)		− 0.07	0.11	-0.18	0.16

Statistically significant values are in bold.

### Influence of other measured ocular characteristics on glare perception

In addition to MPOD, the participants’ photostress recovery time and iris thickness were also measured in experiment-I under color-neutral conditions. Though the effect of photostress recovery time or iris thickness on discomfort glare has not yet been explored in previous research, some studies^8,12^ have tried to relate them to MP and found that patients with a higher MP did seem to have a shorter recovery time; iris thickness, however, was only found to weakly correlate with denser MP. Based on these findings, we formulated the hypothesis that participants with shorter recovery times and denser iris would be better able to tolerate discomfort glare.

From Table 4, we can infer that both photostress recovery time and iris thickness have no statistically significant association with glare perception, independently of the question used to assess it. Additionally, eye strain (question 6, Table 6) was not found to correlate with any of the measured ocular parameters (Table 4). This was further confirmed by a pairwise comparison test on both recovery time and iris thickness between the less sensitive and more sensitive groups defined previously. We again did not find any significant differences between the two groups though we did find a moderate correlation between photostress recovery time and MPOD (Pearson’s rho = 0.40), as would be expected based on literature^8,9^.

## Discussions

Unlike most past studies, we did not find an influence of MP on discomfort glare sensitivity under neutral daylit conditions. One potential explanation could be that the glare source was not restricted to the fovea in our study (mean viewing angle 32°), whereas in previous studies the sources were within at most 6° of the participants' central line of sight^8,9,12,19,21^. Another explanation could be that daylight filtered by neutral glazing was of a broad enough spectrum not to be dominated by short wavelength radiation, and that this was not the case in most of the previous studies that used a shortwave-dominated light source where MP has maximum absorption. To investigate the second explanation further, we exposed participants to red, green, blue, and color-neutral tinted glazing in experiment-II and found a significant correlation between the MPOD and participants’ glare perception only with the blue glazing, whereas like in experiment-I, this correlation became non-significant with the other colored glazings. Therefore, our findings highlight that the spectral composition of the glare source plays a key role in determining the extent to which MP can contribute to discomfort glare protection. Although the color-neutral filters we used in our study had relatively higher transmission under shorter wavelengths compared to regular non-tinted glazing (Fig. 7), it was not as high as the blue-colored filter that had peak transmission at 440 nm, which is strongly absorbed by MP. Consequently, the light received at eye level had higher short-wavelength content in case of blue glazing conditions compared to color-neutral conditions where the contribution of middle- and long- wavelength light was higher (Fig. 2d and f). This indicates that an influence of MP on glare protection would presumably only be seen when using a glare source dominated by short-wavelength radiation.

Another important point relates to the fact that our participants were free to look around, unlike in previous studies. The mean viewing angle between the sun and the participants’ central line of sight (when looking at their monitor screen) was about 32° in the vertical plane, and we estimated that over the course of each experimental session, participants’ average gaze direction varied from + 10° to − 15° in vertical direction based on the recording of their faces during the exposure processed in a deep learning model named OpenFace^40^. However due to certain limitations of the model, it could not accurately predict gaze direction in many scenarios which led to the removal of a substantial amount of gaze data (~ 58%). This gaze behavior indicates that though the glare-source (sun) was not projected in the fovea, there may have been instances where the sun was closer to the fovea and therefore, where the attenuation through the macula may have been stronger. Looking at other studies with relatively free viewing conditions like Stringham et al.^8^, which did find a strong inverse correlation between glare ratings and MPOD (ρ = − 0.60) had used a LED to create glare with a narrow emission spectrum and a large peak at 440 nm and had glare source projected at central 5° of subject’s retina, which are the main differences from the present study. We hypothesized that a smaller viewing angle between the sun and participants’ retina could result in a stronger impact of MP in protection against glare from the sun. Furthermore, the changes in spatial characteristics of the retinal image in central vision, specifically in the image contrast could be hypothesized to contribute more to discomfort glare perception under tested conditions.

One limitation of our study is that its outcomes are based on young & healthy adults (ages 18–30 years), which is not representative of a general workplace population. Therefore, the results should not be extrapolated to individuals of higher age groups and/or with certain eye pathologies since these factors can impact the visual function and affect glare sensitivity^41–45^. It should also be noted that the findings from this study are only valid for the daylit conditions (and spectra) and for the viewing positions experienced by the participants and should not be generalized at this stage. Another aspect worth noting is that, as a result of the rather strict exclusion criteria set for study participation and subsequent post-recruitment discarding of data associated with ocular abnormalities, we ended up with a population sample exhibiting a greater homogeneity compared to past studies, which may conceivably influence some of our study’s outcomes.

Overall the findings demonstrate that MPOD cannot account for the inter-individual variability in discomfort glare sensitivity found in normal working scenarios (i.e. with free gaze behavior, glare source outside the fovea) under neutral daylight conditions, but possibly can, in part, explain the variability when glare is perceived under saturated blue-colored glazing even when the light source may not be in the fovea. This finding should be further confirmed under electrochromic glazing that exhibits blue color and is more widely used in buildings compared to the saturated blue glazing used in our experiment and therefore has higher practical relevance.

The results can also be useful for the development of the glare prediction algorithms that aim to model the optical pathways involved. For such a model to enhance the reliability and accuracy, it could be beneficial to incorporate different range of MP density, particularly when dealing with the glare sources that are located close to the fovea and exhibit a dominant emission in the shorter wavelength. Consequently, the results also provide the direction for the future studies and possible alternate hypotheses which is to investigate the impact of visual pathways beyond the pre-receptoral filters, specifically the possible influence of ipRGCs on glare in short-wave dominated lighting conditions.

## Method

### Study design

The two independent experiments with 55 participants each were conducted in Lausanne, Switzerland (46°31′00.4″ N, 6°33′47.1″ E) during the winter months (Nov’20–Mar’21 and Nov’21–March’22) to benefit from low sun angle. Both experiments followed the same psychophysical approach, where the relationship between the participants’ measured ocular parameters and their subjectively assessed glare sensitivity is investigated. In experiment-I conducted under color-neutral daylit conditions, each participant took part in two test sessions of two hours each: (i) first session on the EPFL (Ecole Polytechnique Fédérale de Lausanne) campus where the participants’ subjective perception of discomfort glare from daylight and electric light was assessed; (ii) second session at the HOJG (Hôpital Ophtalmique Jules Gonin) where the participants had some of their structural and functional ocular parameters measured. In experiment II under blue-tinted daylit conditions, participants took part in a single session at the EPFL campus, which followed the same protocol as the first session of experiment-I. All tests at EPFL were conducted on clear sky sunny days with stable weather so as to use the sun as a glare source visible behind the glazed façade. The tests at HOJG for experiment-I were scheduled according to the availability of the staff and the equipment.

### Ethics

The project protocol was approved by the cantonal ethics commission of Canton Vaud, Switzerland Commission Cantonale d’éthique de la recherche sur l’être humain (CER-VD, ref. No. 2020-00667). We confirm that all methods were performed in accordance with the principles of the Declaration of Helsinki. Participants gave written informed consent before the experiments and were compensated as per the local regulations.

### Participants

We recruited a total of 110 participants in the two experiments based on a pre-selection questionnaire that defined the eligibility criteria. These were to: be in healthy conditions, not be diabetic, have normal color vision, have no other visual impairment, have a BMI within the normal range, and have no extreme chronotypes (chronotype assessed using Morning-Evening Questionnaires^46^), not use drugs and not abuse of alcohol, and be aged between 18 and 35 years. Since all these parameters can impact participants’ indoor comfort perception, we followed them strictly. Other criteria were to have an English proficiency level C1 or higher since the language of instructions and questionnaire was English. To avoid demand characteristics, an additional exclusion criterion was to be from a discipline related to the investigated field (i.e., architecture and civil engineering) or to have a link to the researchers’ topic or the laboratory. Table 5 summarizes the participants’ resulting demographics (number, age, gender, iris color, and vision correction).

**Table 5:** Demographics of the participants.

Experiment	No. of participants	Age (in years)	Sex	Vision correction	Iris color (self-reported)
Experiment I	55	Min = 18 Max = 34 Mean = 23	72% male 28% female	64% no correction 36% glasses or lenses	18% black 22% blue/green/grey 60% brown/hazel
Experiment II	55	Min = 18 Max = 30 Mean = 22.6	71.5% male 28.5% female	53.6% No correction 46.4% glasses or lenses	19.6% black 26.8% blue/green/grey 53.6% brown/hazel

### First session at EPFL (Experiments I & II)

#### Test room setup

**Figure 5:**
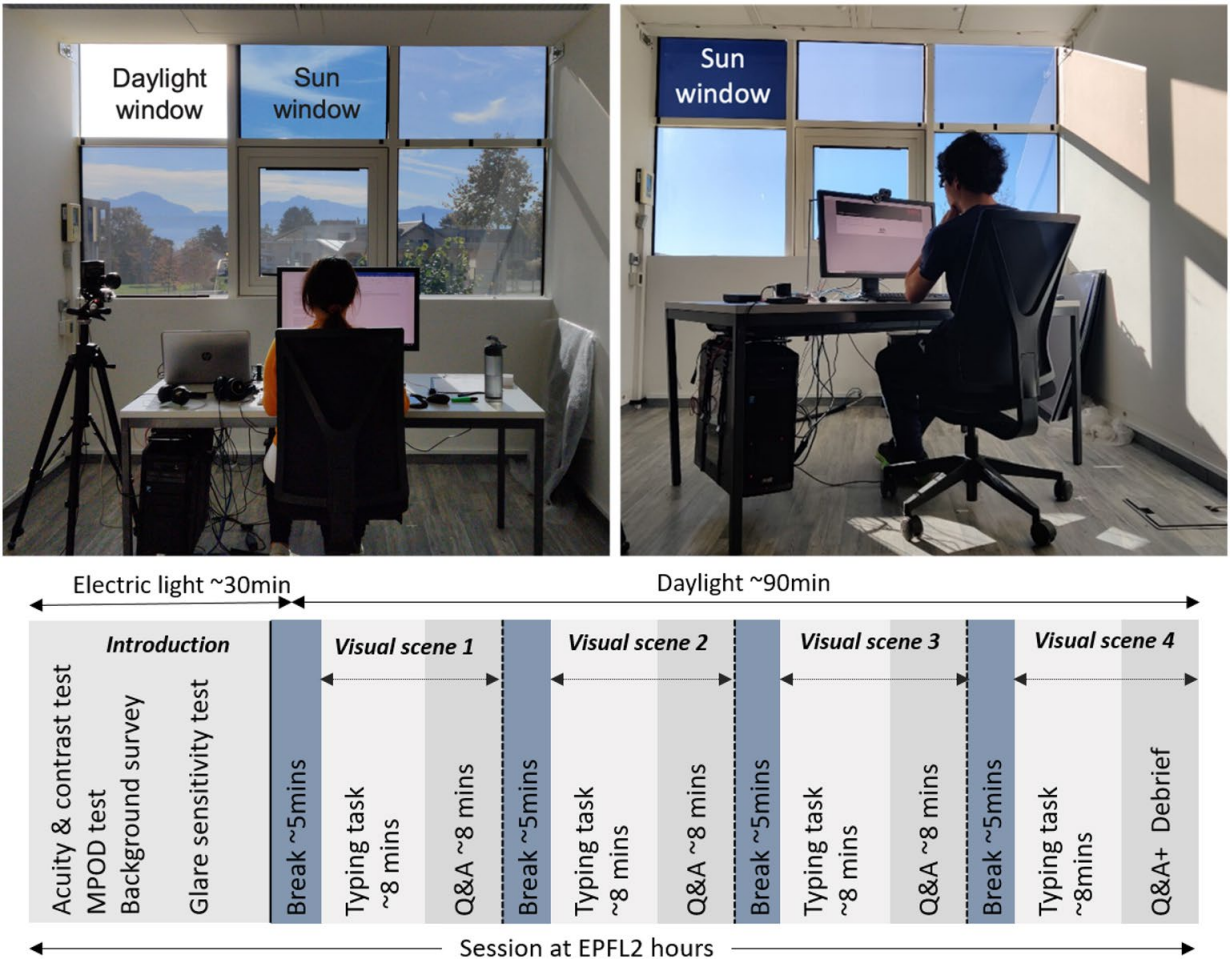
Participant performing the test, left: in experiment I under color-neutral glazing; right: in experiment II under blue-colored glazing (Top), Test procedure at EPFL (Bottom).

The participants’ sensitivity to glare from daylight and electric light was evaluated at the EPFL campus in an office-like test room (3.05 m × 6.55 m), shown in Fig. 5. The test room is fully equipped to control and measure indoor visual and thermal parameters and was used in previous user studies related to comfort^36,47,48^. The room temperature was continuously measured and monitored to keep it within comfortable ranges (21 ± 2 °C) using an indoor climate meter equipped with temperature, humidity, airflow, and CO_2_ sensors. We measured the visual parameters of the room including horizontal illuminance at the desk and vertical illuminance at eye level using lux sensors and measured the daylight spectra at eye level using a spectrometer. We used a calibrated luminance camera LMK 98-4 with a fisheye lens (type Dörr Digital Professional DHG, equidistant projection) to capture high dynamic range (HDR) images of all the experimental conditions from each of the participants’ viewpoints before and after their exposure to the condition. To capture the sun without any pixel overflow, we used neutral density filters ND4 (factor 9366) in experiment-I and a combination of two ND1.8 filters (with a combined factor ND3.5 3134) in experiment-II. A handheld illuminance sensor was mounted below the lens of the camera to compare collected data to the illuminance values derived from the HDR images. Images were captured using the Labsoft software provided with the LMK camera.

#### Test protocol

The overall experimental protocol pertaining to the EPFL session is summarized in bottom part of the Fig. 5 and was the same in both experiments I and II. Experiments were conducted sometime between 9 and 15 h with one participant at a time present for two hours. The first part of the experiment was conducted under constant electric lighting conditions with closed window blinds (i.e., no access to daylight) and after that, participants were exposed to the four experimental conditions with daylight as the only source of light (all electric lights switched off).

Upon arrival, the participants were briefed about the test protocol by the researcher following a single-blind procedure and were asked to sign their written consent to participate. They did the visual acuity and contrast sensitivity tests using a validated software FrACT 3.10.5^29^ on a computer screen at a distance of 170 cm from the screen to allow the measure of the maximal acuity. The brightness of the computer screen was calibrated once using a luminance meter. Afterward, the participants filled out a background questionnaire answering about their demographics, indoor environmental preferences, and their current physical and emotional states.

The researcher measured the participant’s macular pigment optical density (MPOD) for both their eyes in the foveal region of the retina using a macular pigment screener device QuantifEye MPS II^49^. The device employs heterochromatic flicker photometry (HFP) to identify the equal-luminance point of two flickering lights of different wavelengths (530 nm and 465 nm) and provide an estimate of the blue light absorption by MP. In this test, the participants first did the flicker matches for a centrally fixated target (at 0°) and then for a peripheral target (at ± 6°) over a constant background luminance of 250 cd/m^2^ and at a distance of 17 cm. Participants made flicker matches at two light wavelengths: one at 465 nm (blue light), which is absorbed by the MP, and the other at 530 nm (green light), not absorbed by MP. Participants were instructed to press a button as soon as flicker is detected. There was a short familiarisation test before the main test to check their response to flicker. MPOD values are measured on a scale of 0 to 1, where a lower value indicates a higher level of blue light hitting the macula.

Afterwards, a glare sensitivity test was conducted to determine the participants’ sensitivity to glare from electric light in a psychophysical procedure using a dimmable electric light source (only in experiment I). The details of the test and resulting analyses can be found in another publication^50^. That sensitivity test was followed by a break as shown in Fig. 5: during the break, an eye mask was placed on the participant’s eyes for about five minutes to ensure dark adaptation, while the researcher set the test room up for the next tests, all conducted under daylight.

After the break, the participants were seated facing the glazed façade on the south as shown in Fig. 5 left, with the sun apparent in their central field of view. The participants were exposed to four different daylight conditions, experienced in a randomized order and each preceding a dark-adapted break of approximately five minutes, as shown in Fig. 5. During the exposure to each condition, the participants performed a typing task to adapt to daylight and simulate an office environment. Afterwards, they assessed the discomfort caused by glare based on different glare rating scales through a questionnaire to be filled out on screen. During each between-condition break, participants wore an eye mask while the researcher changed the glazing panel to create the next experimental condition and recorded the current luminous conditions using a lux meter and luminance camera to capture HDR images at the participant’s eye level before and after exposure. These images were later processed to calculate the discomfort glare metrics corresponding to each test condition. At the end of the full experiment, participants answered a debriefing questionnaire to provide their overall impression.

#### Test conditions

The four daylight glare conditions experienced by the participants were achieved by altering the glazing transmittance of the windowpane from where the sun was visible to them (labeled as ‘sun window’ in Fig. 5). The tests with daylight were conducted only under stable weather on the clear sky (sunny) days, to ensure consistent and similar daylight availability between all the collected datapoints. The specifics of the test conditions, especially regarding daylight spectrum and intensity, are described below for experiments I and II.

##### Experiment I

**Figure 6:**
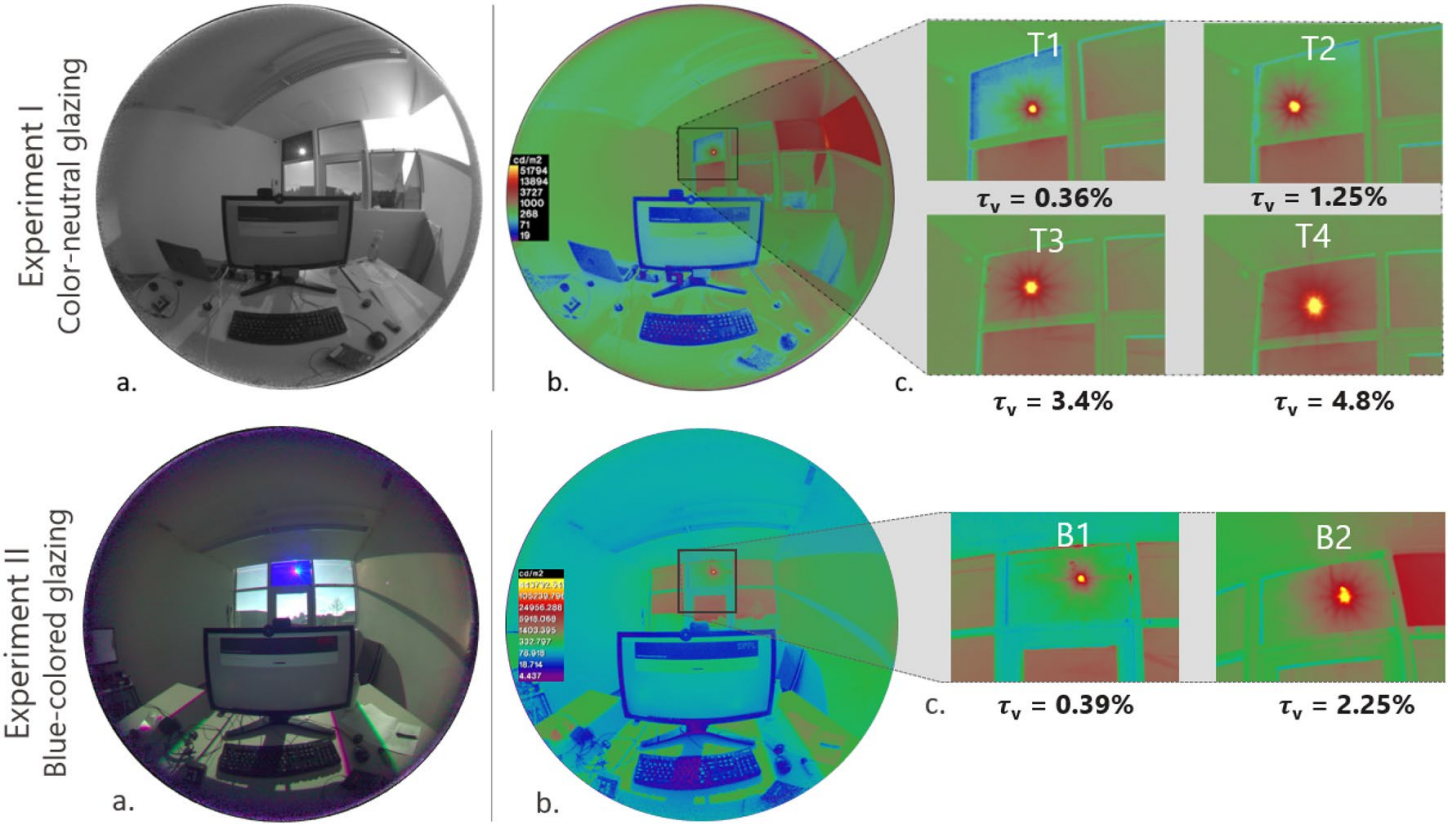
(**a**) Example fisheye images of the test condition in Experiment I and II, (**b**) Falsecolor version of the images showing the scene luminance distribution, (**c**) variation in the sun luminance between the experimental conditions.

**Figure 7:**
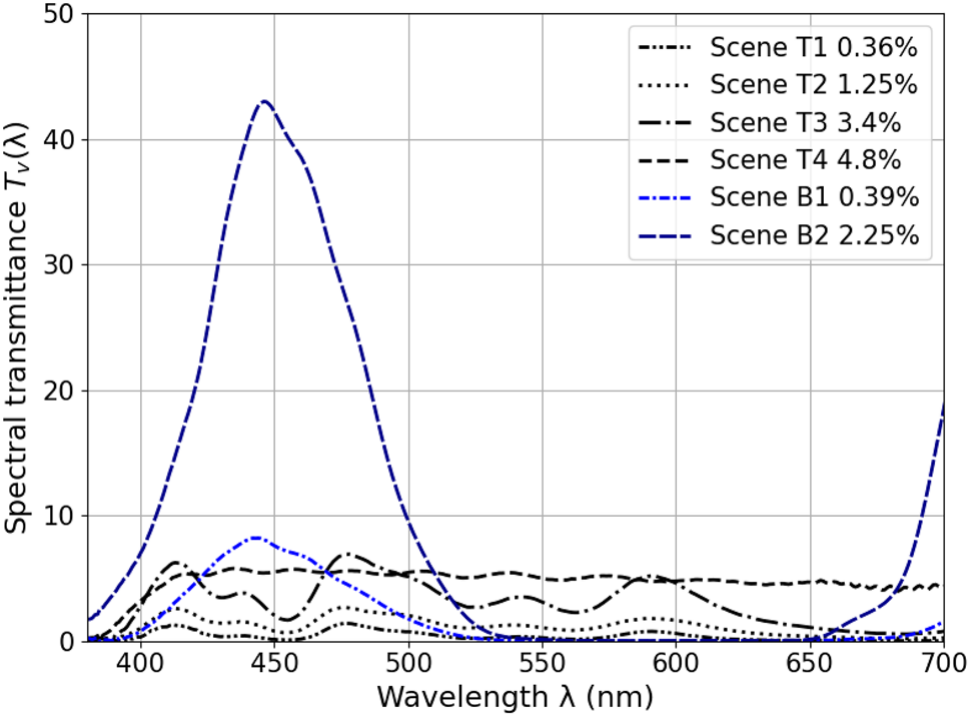
Spectral transmittances of the ‘sun window’ glazing used in experiment I and experiment II.

In experiment-I (Fig. 5), we applied color-neutral films of specific visible light transmittance onto clear acrylic sheets (τ_v,_ = 95%) that were manually fixed against the windowpane (double-glazed window with low-e coating) of the south façade to vary the transmittance of the sun window and thus create the different test conditions. The four conditions under color-neutral tints are referred to as T1, T2, T3, and T4, with measured normal-hemispherical sun window transmittance (τ_v,n–h_) of 0.36%, 1.25%, 3.4%, and 4.8%, respectively. One windowpane, labeled ‘Daylight window’ in Fig. 5 (Top left), was kept at its maximum transmittance of 79% under all four conditions to achieve sufficient daylight levels in the room i.e. at least 300 lx at the desk, which is deemed suitable for office work^51^. The other four windowpanes were kept at a constant transmittance of 4.8%, chosen to avoid glare risks while at the same time maintaining a clear view to the outside. As a result of this setup, the four created conditions, shown as fisheye HDR images and as falsecolor luminance maps in Fig. 6, differed in terms of the luminance of the sun seen through the sun window and the total vertical illuminance reaching the participants’ eyes, thus creating different levels of glare conditions for the participants. To maintain the same viewing direction between the sun and any participant’s view direction in all four conditions, the participant’s desk was rotated as per the sun’s apparent position during the experiment. Figure 7 provides the spectral transmittance of the “sun windows” (combining the film, acrylic sheet, and fixed glazing) used to create the test conditions in experiment I and II. We measured the spectral transmittance of each glazing unit (combination of colored filter and fixed window) and their angular behaviour in a specialized glazing and Nano-tech laboratory by following the setup described by Steiner et al. with a measurement uncertainty of 0.001^52^. Total visible light transmittance, τ_v_ was measured by multiplying the spectral transmittance with the photopic luminosity function V(λ).

##### Experiment II

In experiment II, all participants were exposed to four daylight conditions differing in the color of the glare source, using blue, green, red, and color-neutral glazing, and that were either of extremely low transmittance or of low transmittance. The overall experiment was designed for a different goal that is unrelated to this study, namely to determine the effect of color on glare perception, using two different glare stimuli ranges. In the present study, we will only consider the data collected in the blue glazing conditions, for both the less transmissive sun window glazing (condition B1, sun window τ_v,n–h_ = 0.39%) and the more transmissive one (condition B2, sun window τ_v,n–h_ = 2.25%), whose spectral properties are provided in Fig. 7 together with the color-neutral glazing. Both the B1 and B2 conditions are illustrated in Fig. 6. Similar to experiment-I, only the sun window’s transmission properties were varied between conditions while the remaining windows, all color-neutral, were kept at a constant transmittance of 8% to avoid glare and maintain a clear view as shown in Fig. 5. To make sure that the spectral transmittance of the colored filters was deteriorating due to sun exposure over time, we removed the filters after every experiment and stored them in a dark indoor area.

#### Subjective assessment

During the experiments, participants answered three web-based surveys: (1) a background questionnaire to be filled out at the beginning of the experiment about the participants’ demographics, mood, and indoor environment preferences, (2) a comfort questionnaire provided after exposure to each daylight condition about the visual and thermal comfort, discomfort glare perception, color perception and view out perception, and (3) a debriefing questionnaire provided at the end of the experiment to get an overall feedback on the experiment and the test conditions.

In this section, we go into detail only for the questions from the second type of questionnaire that pertain to discomfort glare or eye fatigue, as these constitute the main focus of the study. Table 6 lists the questions which were analysed in relation to the participants’ ocular measurements (cf. “Results” section): these questions were designed to minimize potential response biases by careful framing of the questions and by using response labels that followed the suggestions and examples found in visual comfort literature^34,47,53^. The order in which the questions were asked was randomized in the survey to avoid any order bias. These questions were answered based on either a binary scale, a Likert (ordinal) scale or linear response labels, or in one case as a free text as shown in Table 6.

**Table 6:** Survey questions and their response labels pertaining to discomfort glare.

Question	Response items
1. Is there anything about the physical environment that disturbs you in this moment?	Open-ended text field
2. Are you experiencing any discomfort due to glare at the moment?	Yes–no
3. At the moment, how would you describe glare in your field of view?	Imperceptible–noticeable–disturbing–intolerable
4. How much discomfort due to glare are you experiencing at the moment?	Not at all–slightly–moderately–very much
5. On a scale of 0–10, how much discomfort due to glare are you experiencing at the moment?	Not at all 0-1-2-3-4-5-6-7-8-9-10 Very much
6. Are you experiencing any eye fatigue or pain on your eyes?	None–slight–moderate–severe

The first survey question was the open-ended question (question 1 in Table 6) that allowed participants to report any disturbance due to the physical environment of the room but without drawing their attention to any specific comfort parameter (such as glare). We evaluated the answers to this question to check if the participants would mention glare spontaneously in their answers since this was the only independent variable varying in the tested conditions. Questions 2, 3, 4, and 5 in Table 6 were asked to inform on the participants’ glare perception under daylight conditions. We asked the same question on different response labels to ensure consistency between a participant’s answers and check the internal reliability of the questions. Question 6 is adapted from the visual functioning questionnaires^54^ to inform on eye fatigue or ocular pain caused by working under each lighting condition and determining its relation with participants’ macular pigment density.

### Second session at HOJG (Experiment I)

In experiment-I, an additional session of ocular examinations was conducted at the ophthalmic hospital to perform an in-depth analysis of various structural and functional aspects of the retina and their relationship with glare sensitivity. After completing the subjective glare assessment test at EPFL, participants visited the HOJG within a couple of days to participate in standard eye exams under the supervision of an ophthalmologist at HOJG. This session took about 2–2.5 h in the late afternoon from 14h30 to 17 h. All participants were first screened by their pathology history, visual acuity, and fundus examination to ensure the absence of ocular pathology. Thereafter they underwent additional structural and functional tests of the macula which include: contrast sensitivity, pupillometry, photostress recovery time, optical coherence tomography, and auto fluorescent fundus imaging as detailed below:

1. Fundus photography autofluorescence (FAF): This test was conducted with Zeiss Clarus widefield digital camera. This photography uses blue light filters in order to detect lipofuscin photopigment abnormalities across the retina. FAF imaging aimed to aid in the documentation and diagnosis of any ocular pathology among the participants.
2. Optical Coherence Tomography (OCT) of iris: This test was conducted using Optovue OCT Model Avanti, Fremont, CA, USA with a laser emission of 840 nm. The iris thickness was estimated from the mean of two cross-sectional images of the anterior segment at 0°–180°, and 90°–270° degree meridians under mesopic conditions for medium pupillary constriction (the pupil was illuminated so as to obtain a diameter of 5 mm in each participant).
3. Pupillometry: Neurolight by IDMed was employed as a portable integrated device to record pupil response to pre-determined light stimuli. This portable integrated device combines a retinal stimulator and pupil recording in a compact instrument using 4 different LEDs and infrared photo video recording at 60 Hz. The pupil response to pre-determined light stimuli was recorded and analyzed to assess outer and inner retinal photoreceptor activity. Using light stimuli in different wavelengths, the different photoreceptors (rods, cones, and melanopsin) are targeted.
4. Contrast sensitivity (2.5%): This test measures visual acuity under low contrast conditions at a contrast of 2.5%. Participants were scored based on the reading of a Pelli-Robson precision vision chart to ensure normal vision.
5. Photostress test: This test measures the time taken by the participant to return to their normal visual acuity following 10 s of bright light exposure.

### Data cleaning and processing

We established data filtering criteria to ensure the reliability and homogeneity of the collected photometric data and to ensure the absence of any ocular pathologies among the participants. Following are the filtering criteria and procedure carried out to clean the data:

1. We discarded the test cases where the deviation in measured on-site global horizontal irradiance (GHI) was more than 25% ((GHI_max_ − GHI_min_)/GHI_mean_) to ensure stable daylight conditions during the entire exposure time and no intermittent clouds occluding the sun.
2. We discarded test cases where the sun was hidden from the participant’s FOV by the window frame or by other elements by manual inspection of the HDR images taken from the participant’s eye position to ensure that the sun stay in the user’s FOV as a glare source.
3. Test cases where the HDR images were found overexposed due to a camera error that couldn’t be resolved to obtain accurate luminance maps were discarded.
4. Data from two participants were discarded because the manual qualitative assessment of the images from the participants’ OCT exam revealed, in one case, an abnormal iris profile due to a scar on the iris, and in another case, relative loss of foveal depression.
5. FAF images were graded by two ophthalmologists independently for hyper- or hypo- fundus autofluorescent abnormalities. In case of disagreement, a third ophthalmologist resolved them. Based on the qualitative assessment of FAF images, data from one further participant was discarded where focal abnormalities were found at the temporal periphery in both eyes.

We post-processed the captured HDR images of the experimental scenes to derive glare metrics and photometric quantities from the luminance maps of the images. Scene images were first converted to “. hdr” format from “.pf” (picture float) format. They were inspected for pixel overflow, which was found in 16 cases where the pixels in the sun disc were saturated, and the image-derived vertical illuminance values were substantially lower (> 25%) than the measured ones. To correct these images, we replaced the overflow pixels to match the measured vertical illuminances. These images were then processed in the Evalglare tool^55^, which is part of the Radiance lighting simulation engine^56^, to derive the glare metrics which were used to compare the experimental conditions.

We collected a total of 275 datapoints, of which 220 were from the 55 participants in experiment I, each exposed to four experimental conditions under color-neutral glazing, and 55 from experiment II (one blue-colored glazing condition per participant). After filtering the data, 18.5% of the data was discarded from experiment I and 3.6% from experiment II which left us with 45 participants in Experiment I and 50 participants in Experiment II. Table 7 presents the summary of the final dataset. All the analyses presented in the “Results” section were performed on this cleaned dataset.

**Table 7:** Summary of data cleaning steps and the resulting dataset.

Data filtering step	Experiment I		Experiment II	
	No. of datapoints	Percentage of collected data	No. of datapoints	Percentage of collected data
Initial dataset	220	100%	55	100%
Step 1 and 2: unstable daylight	− 19	− 8.6%	− 5	− 9.09%
Step 3: luminance camera error	− 10	− 4.45%	0	0
Step 4 and 5: ocular abnormalities	− 3 participants (− 12 datapoints)	− 5.4%	0	0
Final dataset	179	81.5%	50	90.9%

### Statistical methods

Descriptive analyses were conducted to characterize the collected photometric and physiological data with the help of scattered box plots, density plots, and stacked bar plots. To determine the influence of MPOD and other measured ocular parameters on glare sensitivity under daylight conditions, we applied several non-parametric tests. We used a pairwise Wilcoxon signed rank test^38^ to compare the median MPOD values between the more sensitive participants and less sensitive participants under each daylight experimental condition. We conducted correlation analyses to determine the association between the glare responses and the ocular measurements. To correlate responses on binary labels with the MPOD, PSRT, and iris thickness, we applied the Point-biserial correlation coefficient, which is applicable when one of the two variables is dichotomous^57^. We applied Spearman’s rank correlation to compare the ordinal glare responses with the ocular parameters^58^. The strength of the correlation between the two variables was determined by Cohen’s effect size thresholds^35^, which consider a correlation coefficient > 0.3 as a moderate effect, and > 0.5 as a strong effect. The internal reliability of participants’ answers to glare questions was checked by applying Cronbach’s alpha where α > 0.9 presents excellent consistency between the questions^32^.

## Data Availability

Data is available from the corresponding author upon request.
